# Supplementary material for: QIL1 mutation causes MICOS disassembly and early onset fatal mitochondrial encephalopathy with liver disease
Source: eLife. 2016 Sep 13;5:e17163. doi: 10.7554/eLife.17163 (PMC5021520; doi:10.7554/eLife.17163)
Supplement: Supplementary file 1. — (A) Clinical and laboratory findings from patients 1 and 2. Nd: non determined; H: hours of life; mo: months of life. 3MGCA: 3-methylglutaconic aciduria. (B) Respiratory chain activities in control and QIL1-defective patients’ fibroblasts. In fibroblasts, patients’ respiratory chain complexes activities were in control ranges. Complex IV activity normalized to citrate synthase was however more than 3 standard deviations lower than control, indicative a partial defect in complex IV. Activities are expressed in nmol/min/mg protein. (C) Respiratory chain activities in control and QIL1-defective patients’ skeletal muscle for patient 1. In muscle, all respiratory chain complexes (I-IV) activities normalized to citrate synthase were defective. Activities are expressed in nmol/min/mg protein. DOI: http://dx.doi.org/10.7554/eLife.17163.008 [file elife-17163-supp1.docx]

**Guarani et al.**

**Supplementary File 1.**

**Supplementary file 1A.**

| **Clinical and biological signs** | **Pt1: H16** | **Pt1: 6 mo** | **Pt2: H56** | **Pt2: 6-12 mo** | **Ref range** |
| --- | --- | --- | --- | --- | --- |
| **Transient neonatal distress** | + |  | + |  |  |
| **Neurological deterioration**  **Further neurodevelopmental delay** |  | +  + |  | +  + |  |
| **Microcephaly** |  | + |  | + |  |
| **Visual impairment** |  | + |  | + |  |
| **Sensorineural hearing loss** |  | + |  |  |  |
| **Liver nodules** |  | + |  | + |  |
| **Blood lactate (mM)**  **L/P** | 6 | 3.7-4.7  20 | 22.7-8.8  28 | 3.7-6.7  13-15 | <2  <15 |
| **pH/HCO3- (mM)** | 7.33/14 | nd/14 | 7.11/<5 | nd-nd/19-22 | 7.38-7.42/18-25 |
| **Glucose (mM)** | 1.5 |  | 1.9 |  | >2.2 |
| **Blood NH3 (µM)** | 116 | 65 | nd | 44-34 | <50 (100) µM  (neonate) |
| **AST (IU/l)** | 90 | 1682 | 93 | 157-211 | <40 |
| **ALT (IU/l)** | 25 | 1025 | 38 | 89-115 | <40 |
| **Total bilirubin (µM)** | 112 | 29 | 30 | 20-7 | <17 |
| **ɣGT (IU/l)** | 500 | 468 | 1172 | 284-157 | <90 |
| **Prothrombin time** | 19% | 54% | 29% | 81-70% |  |
| **Factor V** | 18% | 81% | 30% | nd-68% |  |
| **α-fetoprotein (ng/ml)** | nd | 10,300 |  | 4233-400 | <100 |
| **Blood tyrosine (µM)** | 677 | 36 | 1002 | 57-28 | 39-77 |
| **Blood methionine (µM)** | 41 | 66 | 73 | 469-84 | 17-29 |
| **3MGCA** | + | + | + | + | absent |

**Supplementary file 1B.**

| **Activity** | **CIV** | **CII-CIII** | **G3PDH-CIII** | **CIII** | **CII** | **G3PDH** | **Citrate synthase** |
| --- | --- | --- | --- | --- | --- | --- | --- |
| Control | 14 | 14 | 8 | 57 | 21 | 13 | 35 |
| Patient 1 | 12 | 15 | 9 | 73 | 24 | 14 | 42 |
| Patient 2 | 15 | 19 | 11 | 63 | 26 | 14 | 50 |
| Control  (n) | 9-57 (42) | 7.5-29 (42) | 5.5-14  (35) | 29-105  (42) | 15-30  (41) | 10-21  (36) | 38-128  (13) |

| **Activity ratios** | **CIV/CII-CIII** | **CIV/G3PDH-CIII** | **CIII/CIV** | **CIV/CII** | **Citrate synthase/CIV** |
| --- | --- | --- | --- | --- | --- |
| Control | 1.0 | 1.8 | 4.1 | 0.67 | 2.50 |
| Patient 1 | 0.8 | 1.3 | **6.1** | 0.50 | **3.50** |
| Patient 2 | 0.8 | 1.4 | 4.2 | 0.58 | **3.33** |
| Control (n=14) | 1.02±0.13 | 1.71±0.28 | 4.21±0.55 | 0.67±0.07 | 2.21±0.31 |

**Supplementary file 1C.**

|  | **CI** | **CIII** | **CII-CIII** | **CIV** | **CS** | **CII** | **CI/CS** | **CIV/CS** | **CIII/CS** | **CII/CS** |
| --- | --- | --- | --- | --- | --- | --- | --- | --- | --- | --- |
| Pt 1 | 26 | 64 | 26 | 97 | 239 | 29 | 0.11 | 0.41 | 0.27 | 0.12 |
| Ctrl  (n=89) | 27-53 | 110-185 | 35-55 | 130-195 | 150-250 | 42-60 | 0.19 ± 0.05 | 0.90 ± 0.24 | 0.76 ± 0.25 | 0.29 ± 0.07 |
