# Supplementary material for: QIL1 mutation causes MICOS disassembly and early onset fatal mitochondrial encephalopathy with liver disease
Source: eLife. 2016 Sep 13;5:e17163. doi: 10.7554/eLife.17163 (PMC5021520; doi:10.7554/eLife.17163)
Supplement: Supplementary file 2. — Tables show QIL1 primers used for PCR amplification. DOI: http://dx.doi.org/10.7554/eLife.17163.009 [file elife-17163-supp2.docx]

**Guarani et al.**

**Supplementary file 2.**

QIL1 Primer sequences.

For genomic DNA:

|  | **Sequences 5'->3'** |
| --- | --- |
| gDNA_C19orf70ex2F (forward) | TCGACGGTGCTGCACTA |
| gDNA_C19orf70ex2R (reverse) | CCTCCCAGAGCTGACCA |

For cDNA:

|  | **Sequences 5'->3'** |
| --- | --- |
| cDNA_C19orf70ex2F (forward) | GCAAGAAAGACTACACCTTCC |
| cDNA_C19orf70ex2R (reverse) | GTCTTCAGGTCAGTGGCAG |
